# Spatial separation and bidirectional trafficking of proteins using a multi-functional reporter

**DOI:** 10.1186/1471-2121-9-17

**Published:** 2008-04-02

**Authors:** Soshana Svendsen, Chad Zimprich, Mark G McDougall, Dieter H Klaubert, Georgyi V Los

**Affiliations:** 1 Promega Corporation 2800 Woods Hollow Road, Madison, WI 53711, USA; 2 Promega Biosciences 277 Granada Drive, San Luis Obispo, CA 93401, USA

## Abstract

**Background:**

The ability to specifically label proteins within living cells can provide information about their dynamics and function. To study a membrane protein, we fused a multi-functional reporter protein, HaloTag^®^, to the extracellular domain of a truncated integrin.

**Results:**

Using the HaloTag technology, we could study the localization, trafficking and processing of an integrin-HaloTag fusion, which we showed had cellular dynamics consistent with native integrins. By labeling live cells with different fluorescent impermeable and permeable ligands, we showed spatial separation of plasma membrane and internal pools of the integrin-HaloTag fusion, and followed these protein pools over time to study bi-directional trafficking. In addition to combining the HaloTag reporter protein with different fluorophores, we also employed an affinity tag to achieve cell capture.

**Conclusion:**

The HaloTag technology was used successfully to study expression, trafficking, spatial separation and real-time translocation of an integrin-HaloTag fusion, thereby demonstrating that this technology can be a powerful tool to investigate membrane protein biology in live cells.

## Background

Membrane proteins are encoded by over 25% of all sequenced open reading frames and constitute the majority of known drug targets [1]. Therefore, tools providing a greater understanding of membrane proteins may benefit cell biology research and pharmacological development [2-5]. The advance of methods for labeling proteins by genetic fusion is expanding the understanding of protein function in complex intracellular environments (see recent reviews) [6-8]. Current reporter proteins such as carrier proteins (i.e. peptidyl PCP or acyl ACP), tetracysteine tags (i.e. Fluorescein and Resorufin Arsenical Helical binders), O^6^-alkylguanine-DNA alkyltransferase (AGT), photoactivatable proteins and others (reviewed by Chapman et al) allow more flexibility than originally available with GFP [5,9-12]. However, visualization of multiple pools of the same protein through space and time can still be technically challenging and new options could only benefit this expanding field.

**Figure 1:**
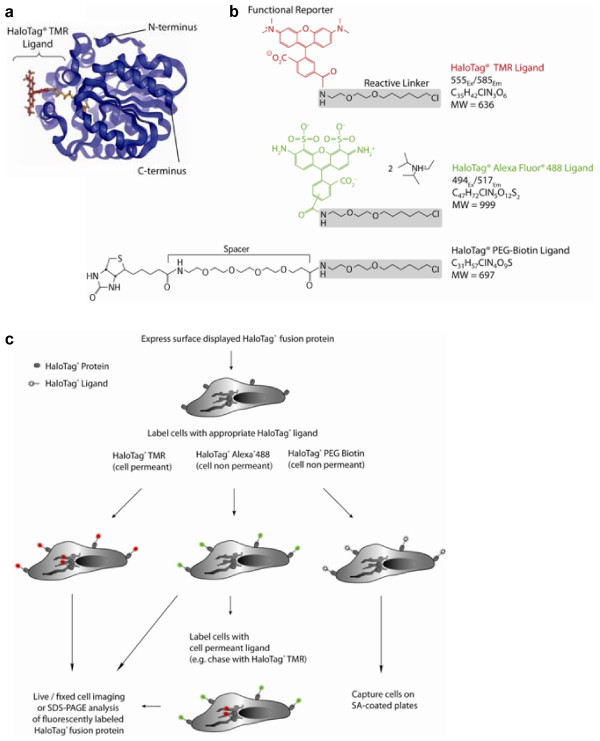
Overview of HaloTag^® ^Technology. **(a) **Molecular model of the HaloTag protein. The HaloTag TMR ligand (fluorescent moiety in red, reactive linker in orange) is shown covalently bound to the aspartate nucleophile (blue). Replacement of catalytic base (His) with Phe renders the HaloTag protein inactive, leading to the formation of a stable covalent bond [13]. **(b) **Chemical structure of the HaloTag ligands showing the functional reporters and the reactive linker. HaloTag ligands have the same chloroalkane reactive linker, but differ in the functional reporter and distance of the reporter from the linker. The HaloTag TMR ligand crosses the cell membrane, unlike the HaloTag PEG-Biotin and Alexa 488 Ligands. **(c) **The interchangeable HaloTag technology permits several cell-based applications, including live or fixed cell imaging and SDS-page analysis. Abbreviations: TMR, tetramethyl-rhodamine; His, histidine; Phe, phenylalanine.

The multifunctional HaloTag^® ^technology complements existing methods and provides new options to study spatial and temporal changes in different pools of a single membrane protein. In addition, it can be used to study protein topology and post-translational modification and to capture cells. The HaloTag technology is based on the formation of a covalent bond between the HaloTag reporter protein and synthetic ligands [13]. The HaloTag reporter protein is an engineered catalytically inactive derivative of a bacterial hydrolase (Figure 1a). The synthetic ligands contain two crucial components: 1) a common reactive linker that forms a covalent bond with the HaloTag protein, and 2) a functional reporter such as a fluorescent dye or an affinity handle such as biotin (Figure 1b). HaloTag ligands have the same chloroalkane reactive linker, but differences in the functional reporter and distance of the reporter from the linker create an interchangeable labeling technology. For instance, the HaloTag^® ^TMR ligand is a cell permeable red-emitting ligand, but unlike some red fluorescent proteins like DsRed, it does not require tetramerization (though directed evolution has since created a monomeric red fluorescent protein) [14,15]. The green-emitting HaloTag^® ^Alexa Fluor^® ^488 and PEG-Biotin are cell impermeable ligands. The interchangeability of a broad range of ligands permits a variety of functional studies of fusion proteins generated from a single genetic construct (Figure 1c).

**Figure 2:**
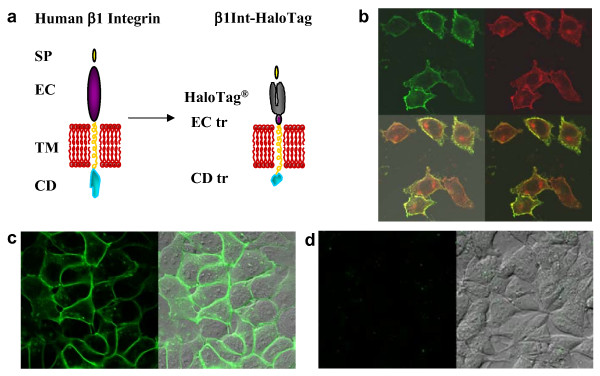
Targeting HaloTag protein to the cell surface. **(a) **Human β1 integrin is depicted with a signal peptide (SP), extracellular domain (EC), transmembrane domain (TM) and cytoplasmic domain (CD) (left). In the β1Int-HaloTag construct, the HaloTag protein is displayed on the cell surface by fusion to the truncated (tr) extracellular domain of human β1 integrin (right). **(b) **Immunocytochemistry, without permeabilization, using antibodies against integrin (green) and HaloTag (red) show that the β1Int-HaloTag fusion protein is being expressed in transfected HeLa Hcells, and the overlap of the two proteins (yellow) indicates β1Int-HaloTag is expressed on the cell surface in a similar pattern to endogenous β1 integrin. **(c) **HEK293 cells stably expressing β1Int-HaloTag labeled with the HaloTag 488 ligand show a green rim, confirming that HaloTag is on the surface and functional. **(d) **HEK293 cells stably expressing HaloTag-(NLS)_3 _labeled with the HaloTag 488 ligand do not show a green rim, confirming that the ligand is cell impermeable and does not label intracellular HaloTag protein. Cell images were generated on an Olympus FV500 confocal microscope using the appropriate filter.

We used an integrin model to assess the applicability of the HaloTag technology for observing the dynamics of membrane protein processing and trafficking. Integrins are membrane proteins that are central to cellular adhesion and migration, thereby involving them in development, inflammation, and disease [16-18]. Integrins are heterodimers of α- and β-subunits, which typically have a large extracellular domain, a single transmembrane segment and a short cytoplasmic tail [16]. Integrins have been studied in living cells by fusing GFP to the intracellular cytoplasmic tail or to the transmembrane domain [19-21]. We fused the HaloTag reporter protein to the extracellular domain of a truncated human β1 integrin (β1Int-HaloTag, Figure 2a). By expressing the HaloTag reporter protein on the cell surface, we were able to use the multifunctional HaloTag technology to study a membrane protein in living cells and to capture cells.

## Results

The β1Int-HaloTag fusion protein was well tolerated by multiple cell types, including mammalian cell lines and human neural stem cells [22]. Immunocytochemistry with β1 integrin and HaloTag antibodies showed that the β1Int-HaloTag fusion protein was expressed at the cell membrane in a similar pattern to endogenous β1 integrin (Figure 2b). Fixed cells were non-permeabilized to show that the HaloTag reporter protein was localized on the cell surface.

To study membrane proteins in live cells with the HaloTag technology, we developed a fluorescent ligand which should not cross the cell membrane. To make this ligand cell impermeable, we added a negatively charged dye to the standard activated linker. To confirm that this novel ligand, HaloTag^® ^Alexa Fluor^® ^488 (HaloTag 488), was cell impermeable, we labeled cells expressing HaloTag on the surface or only inside. Live cell imaging showed that the HaloTag 488 ligand specifically labeled the cell-surface HaloTag protein in cells stably expressing β1Int-HaloTag, but did not label the intracellular protein in cells stably expressing HaloTag fused to a nuclear localization sequence [23] (Figure 2c, d). This confirms that the novel ligand is cell impermeable and that the surface HaloTag protein fused to integrin can functionally bind ligands.

**Figure 3:**
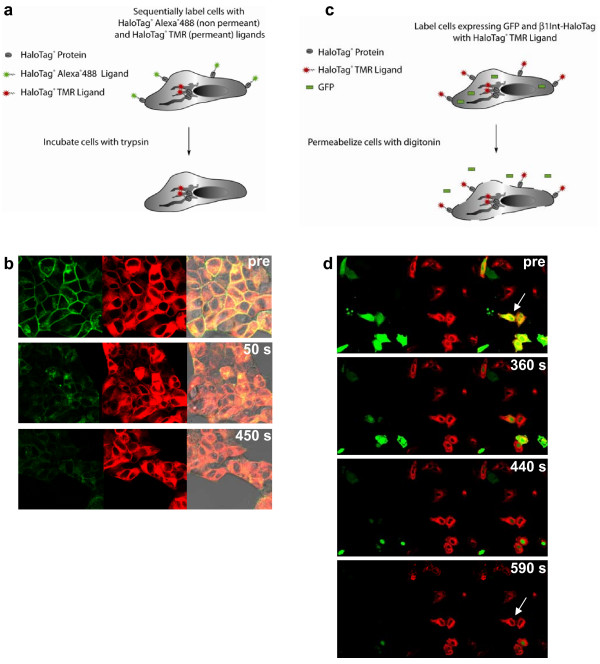
Revealing protein topology and subcellular localization. **(a) **Diagram of separately labeled surface and internal protein pools of β1Int-HaloTag, and trypsin exposure to strip the surface labeled pool. **(b) **Pre trypsin treatment, live HEK293 cells sequentially labeled with HaloTag 488 and TMR ligands show spatial separation of protein pools depicted by a green rim around a red interior. After trypsin, the external HaloTag 488 ligand is stripped over 50 and 450 seconds and the internal HaloTag TMR ligand is preserved. **(c) **Diagram of β1Int-HaloTag and GFP co-expression in TMR-labeled cells, and digitonin exposure to permeabilize membrane. **(d) **HeLa cells transfected with β1Int-HaloTag and GFP were labeled with HaloTag TMR ligand. Co-expression of the 2 proteins, shown by yellow overlay (arrow), is present pre digitonin treatment, but after digitonin most GFP diffuses out of the cells over 360, 440 and 590 seconds and β1Int-HaloTag remains inside (arrow). Cell images were generated on an Olympus FV500 confocal microscope in sequential mode using appropriate filter sets.

To reveal the β1Int-HaloTag protein topology and subcellular distribution, we used HaloTag ligands with a modified fluorescence protease protection (FPP) assay [24]. The FPP assay, described by Lorenz (2006), determines the topology and localization of proteins in living cells by monitoring trypsin-induced destruction of GFP attached to a protein of interest. We separately labeled surface and internal protein pools of β1Int-HaloTag in live cells with the cell impermeable fluorescent ligand, HaloTag 488, followed by the cell permeable fluorescent ligand, HaloTag TMR (Figure 3a). Spatial separation of protein pools is depicted by a green rim around a red interior (Figure 3b). Trypsin exposure to live cells stripped the external HaloTag 488 ligand over time, but preserved the internal HaloTag TMR ligand (Figure 3a, b). This result shows that the HaloTag protein fused to integrin was orientated on the surface of the cell membrane, and that the multi-functional HaloTag technology can be used to determine topology of membrane proteins. In some instances, β1Int-HaloTag labeled on the surface with the HaloTag 488 ligand was internalized before trypsin addition. Unlike the surface exposed protein removed by trypsin, this recycled protein showed fluorescence protease protection (Additional Figure 1).

To reveal the β1Int-HaloTag protein subcellular localization, we combined the HaloTag technology and the permeabilization agent digitonin [24]. Cells were co-transfected with β1Int-HaloTag and GFP, and then labeled with the HaloTag TMR ligand (Figure 3c). Co-expression of the proteins is shown by the yellow overlay (Figure 3d). Digitonin treatment permeabilized the membrane, which allowed freely floating GFP to diffuse out of the cell over time while internally bound β1Int-HaloTag was retained in the cell, presumably by cellular transport and recycling machinery (Figure 3c, d). This result shows that the HaloTag fusion did not affect normal processing of a membrane bound protein, and that the HaloTag technology can be used to distinguish free floating and membrane bound proteins.

**Figure 4:**
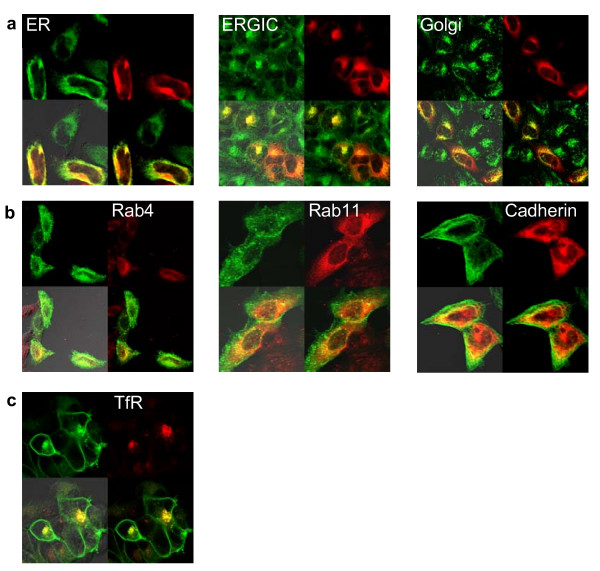
Trafficking and internalization of membrane proteins. **(a) **HeLa cells transiently expressing β1Int-HaloTag were labeled with the HaloTag TMR ligand, then fixed and processed for immunocytochemistry to visualize the endoplasmic reticulum (ER), ER-golgi intermediate complex (ERGIC), or golgi followed by a green secondary antibody. **(b) **HeLa cells transiently expressing β1Int-HaloTag were labeled with the HaloTag 488 ligand, then fixed and processed for immunocytochemistry to visualize early endosomes (Rab4), late endosomes (Rab11) or cadherin followed by a red secondary antibody. **(c) **Hela cells transiently expressing β1Int-HaloTag were labeled with the HaloTag 488 ligand and an Alexa Fluor^® ^594 transferrin conjugate then fixed. Cell images were generated with Olympus FV500 confocal microscope in sequential mode using appropriate filter sets.

Membrane proteins, such as integrins, are typically trafficked through secretory and endocytic pathways [25-27]. Permeabilization experiments showed β1Int-HaloTag was retained in the cell, but to confirm that the β1Int-HaloTag fusion protein was associated with intracellular transport and recycling organelles, we combined fluorescent HaloTag ligands to label live cells with fixed cell immunocytochemistry. To assess protein delivery to the membrane, live cells expressing β1Int-HaloTag were labeled with the HaloTag TMR ligand, and then processed for immunocytochemistry to visualize the endoplasmic reticulum (ER), ER-intermediate golgi complex (ERIGC), or golgi (Figure 4a). To assess protein recycling from the membrane, live cells expressing β1Int-HaloTag were labeled with the HaloTag 488 ligand, and then processed for immunocytochemistry to visualize the early and late endosomes (Figure 4b). Co-localization of the β1Int-HaloTag protein with cellular transport machinery is shown by the yellow overlay, and suggests that fusing HaloTag to the truncated integrin does not alter normal protein flow through secretory and endocytic pathways.

We used the HaloTag technology and immunocytochemistry to also show that the β1Int-HaloTag protein co-localized with expected membrane proteins, such as cadherin and transferrin receptors [20,28,29]. To assess protein co-localization at the membrane, live cells expressing β1Int-HaloTag were labeled with the HaloTag 488 ligand, and then processed for immunocytochemistry (Figure 4b). Co-localization of the β1Int-HaloTag protein with cadherin is shown by the yellow overlay, and suggests that β1Int-HaloTag fusion not only traffics properly to the membrane but also co-localizes with other expected membrane proteins. To assesses protein co-internalization from the membrane, live cells expressing β1Int-HaloTag were labeled with the HaloTag 488 ligand and a transferrin Alexa Fluor^® ^594 conjugate (Figure 4c). Co-localization of the β1Int-HaloTag protein with the transferrin receptor is shown by the yellow overlay, and suggests that the β1Int-HaloTag fusion not only internalizes through the proper cellular machinery, but also co-internalizes with expected membrane proteins.

**Figure 5:**
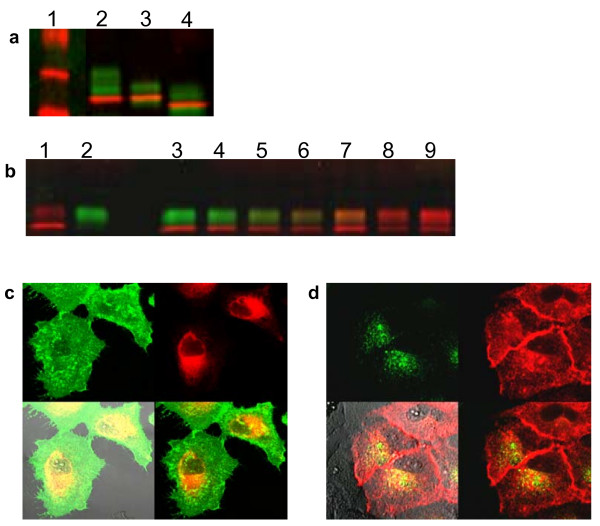
Spatial and temporal separation of proteins using HaloTag Technology. **(a) **HEK293 cells stably expressing β1Int-HaloTag were sequentially labeled with the HaloTag 488 and TMR ligands and then rinsed and lysed. Lysate was non-glycanase treated (lane 2), O-glycanase treated (lane 3) or N-glycanase treated (lane 4) and analyzed by SDS-PAGE with a ladder (lane 1). **(b) **HEK293 cells stably expressing β1Int-HaloTag were labeled with the HaloTag TMR ligand alone (lane 1), the HaloTag 488 ligand alone (lane 2), or sequentially with the HaloTag 488 and TMR ligands (lanes 3–9). Cells were rinsed and lysed at 0 minutes (lanes 1–3) or 0.5, 1, 2, 4, 8 and 12 hours (lanes 4–9) after labeling, and lysate was run on SDS-PAGE. **(c) **HeLa cells expressing β1Int-HaloTag were sequentially labeled with the HaloTag 488 and HaloTag TMR ligands and imaged immediately after labeling to show spatially separated protein pools. **(d) **Cells were re-imaged 12 hours after labeling to show temporal translocation of both protein pools. Cell images were generated with Olympus FV500 confocal microscope in sequential mode using appropriate filter sets.

Mature integrins at the cell membrane are glycosylated, and the internal pool is partially glycosylated as it traffics through the secretory pathway [30]. To confirm that the β1Int-HaloTag protein not only traffics normally through the secretory pathway but also undergoes proper post-translational modification, we used fluorescent HaloTag ligands followed by SDS-PAGE analysis. Live cells expressing β1Int-HaloTag were sequentially labeled with HaloTag 488 and TMR ligands to separately label surface and internal proteins, respectively (Figure 1c). Lysate from labeled cells showed two distinct protein pools on SDS-PAGE in lane 2 (Figure 5a). This band pattern could be because the higher molecular weight green surface pool of β1Int-HaloTag is heavily glycosylated compared to the red intracellular pool. To confirm whether the distinct protein pools were due to differential protein glycosylation, we glycanase treated cells lysates. Deglycosylation by either O- or N-glycanase treatment caused a significant shift in the green surface pool of β1Int-HaloTag (lane 3 and 4). Conversely, O-glycanase treatment caused no visible shift in the red internal pool of β1Int-HaloTag (lane 3), and N-glycanase treatment caused only a minor shift (lane 4). Glycanase treatment of the HaloTag reporter protein produced no band shift on SDS-PAGE (data not shown), suggesting that the shift in β1Int-HaloTag is due to glycosylation of the integrin protein. Our results show that the surface pool of β1Int-HaloTag is glycosylated, which is expected for integrins and suggests that fusing HaloTag to a truncated integrin does not alter proper post-translational glycosylation.

We also used the HaloTag technology to follow protein modification over time. Cells expressing β1Int-HaloTag were labeled sequentially with HaloTag 488 and TMR ligands, and lysate was then collected immediately or up to 12 hours after labeling (Figure 5b). As expected, lysate from cells labeled with the HaloTag TMR ligand alone showed two protein pools (lane 1) and lysate from cells labeled with the HaloTag 488 ligand alone showed only the higher molecular weight protein pool (lane 2). Substantiating figure 5a, lysate from cells sequentially labeled with both ligands showed separation of the two protein pools at early time points (lanes 3–5). However, over time the lower band for the red internal pool shifted up, presumably as this protein arrived at the membrane in a glycosylated form (lanes 6–9) [31]. Additionally, the upper band for the green surface pool disappeared, presumably as this protein was endocytosed and degraded. The overlaid SDS-PAGE shows that the HaloTag technology can be used to track different protein pools and monitor post-translational modifications over time.

Finally, we used the HaloTag technology and live cell imaging to visualize spatial separation and real-time translocation of β1Int-HaloTag. Cells expressing β1Int-HaloTag were labeled sequentially with HaloTag 488 and TMR ligands. Live cell imaging showed two distinct protein pools, with the surface protein labeled specifically with the HaloTag 488 ligand and the intracellular protein labeled with the HaloTag TMR ligand (Figure 5c). Re-imaging 12 hours after labeling shows that the initial red cytoplasmic pool has moved to the membrane and the initial green surface pool has internalized (Figure 5d). Timelapse imaging shows real-time translocation after labeling, which begins with the green surface pool internalizing at 1 hour, continues with the red internal pool trafficking to the surface, and ends with the red surface pool internalizing (Additional Figure 2). The HaloTag technology can show clear translocation of two separate protein pools in live cells, and these results of β1Int-HaloTag movement corroborate the immunocytochemistry and SDS-PAGE results.

**Figure 6:**
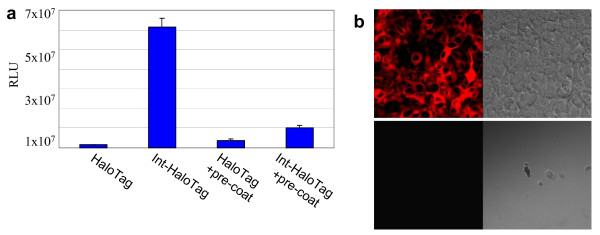
Cell capture using HaoTag Technolgy. **(a) **HeLa cells co-expressing β1Int-HaloTag and luciferase were labeled with HaloTag PEG-Biotin. They show specific capture on SA plates compared to labeled cells co-expressing HaloTag and luciferase. Pre-coating plates with HaloTag PEG-Biotin blocks the specific capture. **(b) **HEK293 cells stably expressing β1Int-HaloTag (top panel) or wildtype HEK293 (bottom panel) were labeled with HaloTag PEG-Biotin. After capture and replate, cells were Tamra labeled and live cell imaging shows β1Int-HaloTag-expressing cells are specifically captured and survive.

With the HaloTag technology, functional reporters such as fluorescent ligands can be used to image live cells, and affinity handles such as biotin can be used to capture cells expressing HaloTag on the surface. We co-expressed β1Int-HaloTag, or HaloTag as a control, with luciferase. Live cells were labeled with HaloTag PEG-Biotin ligand and then captured on streptavidin coated plates. Luciferase assay results show the specific capture of β1Int-HaloTag-expressing cells compared to HaloTag-expressing control cells (Figure 6a). Specific capture of β1Int-HaloTag-expressing cells was blocked when streptavidin coated plates were pre-coated with HaloTag PEG-Biotin. In addition to the *in vitro *luciferase assay, live cell imaging confirms that β1Int-HaloTag-expressing cells can be specifically captured using the HaloTag technology and that captured cells survive. Live cells were labeled with HaloTag PEG-Biotin ligand, captured on streptavidin coated plates, and then replated for live cell imaging. Labeling plated cells with the HaloTag TMR ligand shows the survival of specifically captured β1Int-HaloTag-expressing cells compared to control cells (Figure 6b).

## Discussion

The ability to analyze proteins in their native environment is critical to developing a detailed understanding of protein processing and trafficking. The study of protein trafficking is particularly timely and valuable considering the recently reported link between disrupted protein trafficking and certain disease states [32,33]. The study of protein glycosylation patterns is also relevant to disease states, as recently reported by Lyly et al for palmitoyl protein thioesterase I related to childhood encephalopathies [34].

We showed that the HaloTag technology can be used to study the expression, topology, glyscosylation, distribution, and translocation of a vital cellular protein. This was possible in multiple cell types, including mammalian cell lines like CHO, HeLa, HEK293 and U2OS and also in human neural stem cells (data not shown) [22]. The need for antibodies is eliminated since the fixation-resistant fluorescent HaloTag ligand directly detects the HaloTag reporter protein fused to the protein of interest. Efficient and flexible labeling of fusion proteins in live cells enables a fuller understanding of the protein's function.

By fusing the HaloTag gene to the extracelluar domain of a truncated β1 integrin, we provided proof of concept that the HaloTag reporter protein can be expressed on the cell surface and can be used to study various aspects of membrane protein biology. In addition to integrin, we have successfully used the HaloTag technology to study the biology of other membrane proteins, including glycosylphosphatidylinositol (GPI) and the GABA_A _receptor (data not shown). While we can not rule out subtle effects of the HaloTag reporter protein on β1 integrin, we provide strong evidence that integrin function was not affected. Results showing that β1Int-HaloTag localized in a similar pattern to endogenous integrin, trafficked and internalized through expected intracellular machinery and followed proper post-translational glycosylation suggest that the integrin-HaloTag fusion protein maintained cellular dynamics consistent with native integrins.

To specifically study surface-displayed HaloTag fused to membrane proteins, we developed a novel HaloTag Alexa 488 ligand that we showed is cell impermeable and functional. A previous report made an extracellular GFP-integrin fusion and showed impressive focal adhesion motility in live fibroblasts [21]. However, GFP was used primarily as a fluorescent marker of the fusion protein rather than the multifunctional HaloTag-integrin fusion we used to spatially and temporally visualize integrin, to determine integrin topology and post-translational modification and to capture live cells. In addition, GFP was fused directly to the transmembrane domain with complete removal of the extracellular domain. While we truncated the integrin in our β1Int-HaloTag construct, we intentionally retained portions of the extracellular and cytoplasmic sequences and the specificity determining loop (SDL) to ensure integrin traffics to the membrane [35,36].

In addition to using the HaloTag technology to study membrane protein biology, we have applied the HaloTag technology to sort cells by fluorescent activated cell sorting FACS [37]. We were able to successfully separate β1IntHT2-expressing cells labeled with HaloTag TMR or Alexa488 ligands from non-expressing cells (data not shown). As an alternative method for cells sensitive to FACS, the HaloTag technology can also be used to sort cells by panning [38]. We showed that the technology can be used to select cells by labeling surface-expressed HaloTag with the PEG-Biotin ligand. Labeled cells can then be captured on a streptavidin plate for *in vitro *assays or live cell imaging.

Los and Wood (2006) previously described the HaloTag technology simply for imaging intracellular proteins and only using cell permeable ligands. By expressing HaloTag on the cell surface and developing a fluorescent cell impermeable ligand, we have greatly expanded this multifunctional technology to include visualizing different pools of a membrane protein over space and time, assessing post-translational modification, and capturing cells. In addition, we combined the HaloTag technology with FPP and digitonin permeabilization to study protein topology and distribution.

The multifunctional HaloTag technology supports many experimental procedures including immunocytochemistry, fixed and live cell imaging, SDS page, FPP and capture. We successfully used this technology to determine protein topology and subcellular localization, to capture and sort cells, and to assess protein modification. In addition, we used different colored cell impermeable and permeable HaloTag ligands to show spatial separation of membrane and internal protein pools, and real-time translocation of these protein pools in live cells.

## Conclusion

We have shown that the multifunctional HaloTag technology provides the ability to separate protein pools in space and time, and could be a powerful tool to examine the trafficking and cellular biology of membrane proteins such as integrins or other proteins of interest.

## Methods

### HaloTag^® ^protein and ligands

The HaloTag reporter protein is an engineered catalytically inactive derivative of a bacterial hydrolase [13]. Replacement of the catalytic base (His) with Phe renders the HaloTag protein inactive by impairing its ability to hydrolyze the ester intermediate, leading to the formation of a stable covalent bond. The synthetic ligands contain two crucial components: 1) a common reactive linker that forms a covalent bond with the HaloTag^® ^protein, and 2) a functional reporter such as a fluorescent dye or an affinity handle such as biotin. HaloTag^® ^ligands have the same chloroalkane reactive linker, but differ in the functional reporter and distance of the reporter from the linker. The HaloTag^® ^TMR ligand crosses the cell membrane, unlike the HaloTag^® ^PEG-Biotin and HaloTag^® ^Alexa Fluor^® ^488 Ligands.

### HaloTag^® ^ligand synthesis

The succinimidyl ester of Alexa Fluor^® ^488 carboxylic acid was obtained from Invitrogen. Biotin-PEO_4_-propionate succinimidyl ester was purchased from Biotium. All other reagents and solvents for chemical syntheses were purchased from Aldrich, Fisher, or VWR and were used without further purification unless specified. Nuclear Magnetic Resonance (NMR) was recorded on a *Varian*-300. Mass data were obtained using a *Fisions *VG Platform II spectrometer. UV-Vis absorption spectra were recorded on *Beckman *DU 650 in 50 mM phosphate buffer (pH = 7.4). HPLC purification was accomplished using a *Waters *Preparative HPLC with water and acetonitrile (ACN) as mobile phase carriers applied to a *Varian *Microsorb 60-8 C18 column; 250 × 21.4 mm. Analytical HPLC was performed on *Agilent *1100 HPLC using a *Synergi *4 μ Max-RP 80R, 250 × 4.6 mm C12 column with 10 mM ammonium acetate, pH = 5.5 (A) and ACN (B) as mobile phase from 25% to 60% B in 15 minutes. Combustion data was obtained by *Quantitative Technologies, Inc*.

#### 3,6-Diamino-9-[2-carboxy-4(or 5)-[N-2-(2-(6-chlorohexyloxy)ethoxy)ethyl carbamoyl]phenyl]-4,5-disulfo-xanthylium, bis(diisoproplyethylammonium) salt

To a stirring solution of 3,6-diamino-9-[2-carboxy-4(or 5)-[[(2,5-dioxo-1-pyrrolidinyl)oxy]carbonyl]phenyl]-4,5-disulfoxanthylium, inner salt (5 mg, 5.3 × 10^-5 ^mol) in 1 ml dry DMF (stored over molecular sieves) was added via syringe a 2M solution of 2-[2-(6-chloro-hexyloxy)-ethoxy]-ethyl-amine (40 μl, 7.95 × 10^-5 ^mol) in CH_2_Cl_2 _followed by diisoproplyethylamine (46 μl, 2.6 × 10^-4 ^mol). The reaction mixture was allowed to stir for 12 hours then diluted with 1 ml of water and subjected to prep HPLC purification. An orange solid was isolated.

Yield: 21 mg, 68%.

λ_max_: 493 nm; ε: 77,202

HPLC: 10.39 min (63.5%) and 10.70 min (36.5%).

^1^H NMR (D_2_O) [5 isomer]: δ = 8.34 (d, 1H, Ar-4), 8.03 (dd, 1H, Ar-6), 7.45 (s, 1H, Ar-7), 7.26 (d, 2H, Ar-1',8'), 6.97 (d, 2H, Ar-2',7',8'), 3.80 (dd, 1H, exchangable, NH), 3.78-3.50 (m, 10H, CH_2_-O and CH_2_-NH(CO)), 3.73 (M, 4H, CH-NH^+^), 3.42 (t, 2H, CH_2_-Cl), 3.21 (dd, 4H, CH_2_-NH+), 1.69-1.24 (m, H8, -CH_2_-), 1.35 (d, 24H, CH_3_-CH), 1.35 (t, 6H, CH_3_-CH_2_) ppm. [6 isomer]: δ = 8.06 (s, 2H, Ar-4,7), 7.67 (s, 1H, Ar-5), 7.26 (d, 2H, Ar-1',8'), 6.97 (d, 2H, Ar-2',7',8'), 3.80 (dd, 1H, exchangable, NH), 3.78-3.50 (m, 10H, CH_2_-O and CH_2_-NH(CO)), 3.73 (M, 4H, CH-NH^+^), 3.42 (t, 2H, CH_2_-Cl), 3.21 (dd, 4H, CH_2_-NH+), 1.69-1.24 (m, H8, -CH_2_-) ppm, 1.35 (d, 24H, CH_3_-CH), 1.35 (t, 6H, CH_3_-CH_2_) ppm.

MS: *m/z *Calcd for C_31_H_34_ClN3O_12_S_2_^+^: 739.13. Found: 739.30.

EA: Calcd for C_47_H_72_ClN_5_O_12_S_2_: C 56.52, H 7.27, N 7.01, S 6.42, Cl 3.55. Found: C 58.66, H 6.85, N 7.16, S 6.84, Cl 4.62.

#### N-(2-(2-(6-chlorohexyloxy)ethoxy)ethyl)-1-(5-((3aS,4S,6aR)-2-oxo-hexahydro-1H-thieno [3,4-d]imidazol-4-yl)pentanamido)-3,6,9,12-tetraoxapentadecan-15-amide

To a stirring solution of biotin-PEO_4_-propionate succinimidyl ester (5 mg, 8.5 × 10^-6 ^mol) in 115 μl dry DMF was added via syringe a 0.3M solution of 2-[2-(6-chloro-hexyloxy)-ethoxy]-ethyl-ammonium chloride (85 μl, 2.55 × 10^-5 ^mol) in CH_2_Cl_2 _followed by one drop of diisoproplyethylamine (excess). The reaction mixture was allowed to stir for 4 hours then diluted to 1 ml of water and subjected to prep HPLC purification. A white solid was isolated.

Yield: 4.2 mg, 71%.

^1^H NMR (DMSO-d6) [5 isomer]: δ = 7.88 (t, 1H, exch., NH), 7.82 (t, 1H, exch., NH), 6.41 (s, 1H, exch.., biotin-NH), 6.34 (s, 1H, exch., biotin-NH), 4.30 (m, 1H, CH), 4.12 (M, 1H, CH), 3.62 (t, 2H, CH_2_-Cl), 3.58 (t, 2H, CH_2_-O), 3.50-3.30 (m, 24H, CH_2_), 3.18 (m, 4H, CH_2_-N), 3.08 (m, 1H, CH), 2.82 (dd, 1H, CH_2_), 2.57 (d, 1H, CH_2_), 2.31 (t, 2H, CH_2_), 2.06 (t, 2H, CH_2_), 1.75-1.27 (m, 12H, -CH_2_-) ppm.

MS: *m/z *Calcd for C_31_H_58_ClN4O_9_S^+^: 697.36 (100%), 698.36 (35.8%), 699.36 (37.3%). Found: 697.64, 699.68.

EA: Calcd for C_31_H_57_ClN_4_O_9_S: C 53.39, H 8.24, N 8.03, S 4.60, Cl 5.08. Found: C 53.08, H 8.45, N 7.82, S 4.31, Cl 5.03.

TLC (10% MeOH in CH_2_Cl_2_, PMA stain): 0.33

### Construct design

The HaloTag mammalian expression vector (pHT2, Promega) contains the open reading frame of the modified hydrolase gene, which contains a 5' BamHI and 3' NgoMIV restriction site. The human β1 integrin sequence was synthesized (Blue Heron) with a signal peptide (amino acids 1–20), a truncated extracellular domain (amino acids 190–217, 722–730), a transmembrane domain (amino acids 731–756), and a truncated cytoplasmic domain (amino acids 757–762). The specificity determining loop was retained in the extracellular domain [36]. Using the BamHI and NgoMIV sites, HaloTag was cloned downstream of the signal peptide and upstream of the extracellular domain. The HaloTag protein was also cloned upstream of three copies of a nuclear localization sequence (NLS)_3 _from the simian virus large T-antigen [23]. The β1Int-HaloTag and HaloTag-(NLS)_3 _fusion proteins were confirmed by restriction digest analysis and sequencing. DNA was purified using endofree maxipreps (Qiagen) for transfection into mammalian cell lines.

### Cell culture and transfection

All procedures were performed at the standard cell growing conditions of 37°C in an atmosphere of 5% CO_2_. HeLa (ATCC #CCL-2), CHO-K1 (ATCC #CCL61), HEK293 or U2OS (ATCC #HTB-96) cells were maintained in DMEM, F12 or McCoy 5A media supplemented with 10% fetal bovine serum (Invitrogen, USA) according ATCC recommendations.

The β1Int-HaloTag fusion protein was transfected into mammalian cell lines using the cell-specific lipofection reagents from Mirus, according to manufacturer protocols. Co-transfection experiments using β1Int-HaloTag and humanized renilla luciferase or monster GFP (Promega) were done at a 1:1 ratio. Cells stably expressing β1Int-HaloTag or HaloTag-(NLS)_3 _were generated using a 3:1 ratio of β1Int-HaloTag or HaloTag-(NLS)_3 _and pcINeo (Promega).

### Microscopy

Cells were imaged on a confocal microscope FV500 (Olympus, Japan) using a 488 nm Ar/Kr laser line or a 543- or 633-HeNe laser line. Scanning speed and laser intensity were adjusted to avoid photobleaching of the fluorophores and damage of the cells. The microscope was equipped with microenvironmental chamber to maintain physiological conditions.

### Cell labeling

Live cells were labeled for 15 minutes at 37°C with HaloTag^® ^TMR (5 μM), HaloTag^® ^Alexa Fluor^® ^488 (5 μM), or HaloTag^® ^PEG-Biotin (10 μM) ligands. Cells were labeled with individual ligands, or sequentially labeled first with the HaloTag 488 ligand followed by the HaloTag TMR ligand. The cell permeable HaloTag TMR can label the entire pool of HaloTag protein and the cell impermeable HaloTag 488 ligand can label only surface protein. After labeling, cells were rinsed with fresh media for 15–30 minutes. Cells were then imaged live, or fixed and processed for immunocytochemistry, or lysed and processed for SDS-page analysis.

### Live cell imaging

To confirm surface expression and functionality of the HaloTag protein, HEK293 cells stably expressing β1Int-HaloTag or HaloTag-(NLS)_3_were labeled with the HaloTag 488 ligand, then rinsed and imaged. To assess spatial separation of proteins in live cells using the HaloTag technology, HeLa cells were transiently transfected with the β1Int-HaloTag protein, and 48 hours later cells were sequentially labeled with the HaloTag 488 ligand followed by the HaloTag TMR ligand. After labeling and rinsing, cells were imaged immediately and 12 hours later. To assess real-time translocation of proteins in live cells using the HaloTag technology, U2OS cells stably expressing the β1Int-HaloTag protein were sequentially labeled with the HaloTag 488 and TMR ligands, then rinsed, and imaged immediately followed by every 20 minutes for 17 hours.

### Fixed cell imaging

The stability of the covalent bond between the HaloTag protein and the HaloTag ligands and the resistance of the fluorescent signal to cell fixatives allows the imaging of β1Int-HaloTag in fixed cells. HeLa cells were transiently transfected with the β1Int-HaloTag protein, and 48 hours later cells were labeled with either the HaloTag 488 or TMR ligand. For transferrin experiments, cells were simultaneously labeled with HaloTag 488 and an Alexa^® ^Fluor 594 transferrin conjugate (Invitrogen, per supplier protocol).

Hela cells were fixed with 4% paraformaldehyde for 10 minutes and then rinsed with phosphate buffered saline (PBS). For Rab4, Rab11 and transferrin studies, cells were fixed 30 minutes after live cell labeling to allow time for protein internalization. Cells were blocked in PBS with 5% normal goat serum (Gibco) and 0.1% triton (Sigma), except for double immunocytochemistry with HaloTag^® ^and integrin where triton was omitted so cells were non-permeablized. After block, cells were incubated in the following primary antibodies: HaloTag^® ^(rabbit, 1.5 μg/ml, Promega), integrin (mouse, 1:100), golgi zone (mouse, 1:20, Chemicon), protein disulfide isomerase (PDI) (endoplasmic reticulum marker, mouse, 1:350, Abcam), 58 K protein (endoplasmic reticulum intermediate golgi complex marker, mouse, 1:200, Abcam), rab4 (early endosome marker, mouse, 1:100, Abcam), rab11 (late endosome marker, mouse, 1:200, Abcam), and cadherin (rabbit, 1:600, Abcam). Primary antibodies were removed and rinsed 3 times with PBS followed by Alexa Fluor^®^-488 conjugated or Alexa Fluor^®^-594 conjugated anti-rabbit or mouse secondary antibodies (goat, 1:1000, Molecular Probes). Cells were rinsed 3 times with PBS and imaged.

### Fluorescence protease protection

To reveal the β1Int-HaloTag protein topology and subcellular localization, we used the HaloTag technology with a modified fluorescence protease assay – a new technique that determines the topological distribution of proteins in living cells by monitoring trypsin-induced destruction of GFP attached to a protein of interest [24]. To assess protein topology, HEK293 cells stably expressing the β1Int-HaloTag protein were sequentially labeled with the HaloTag 488 and TMR ligands. Immediately following labeling and rinsing, live cells were imaged before trypsin addition and then imaged at 50 and 450 seconds following 4 mM trypsin exposure. For additional figure 1, live cells were imaged 1 hour following labeling and rinsing, thereby some β1Int-HaloTag that had labeled on the surface with the HaloTag 488 ligand would internalize before trypsin addition. To assess subcellular localization, HeLa cells co-expressing the β1Int-HaloTag protein and GFP (Promega) were labeled with the HaloTag TMR ligand. Immediately following labeling and rinsing, live cells were imaged before digitonin addition and then imaged at 360, 440 and 590 seconds following 80 μM digitonin treatment.

### Cell capture

HeLa cells were transiently co-transfected with β1Int-HaloTag, or HaloTag as a control, and humanized renilla luciferase. After 48 hours, cells were labeled with the HaloTag PEG-Biotin ligand, then rinsed, and collected with 3 mM EDTA. Cells were placed into SA-coated 96 well plates (Pierce) for 30 minutes at 37°C, then wells were rinsed to remove non-captured cells. Captured cells were lysed and luciferase activity was measured using the renilla luciferase assay, per manufacturer protocol (Promega). Plates were read on a microplate luminometer (Veritas) and RLU (relative light units) were averaged over 24 wells, with standard error of the mean. To verify specific capture, SA-coated plates were pre-blocked with the HaloTag PEG-Biotin ligand (10 μM, for 30 minutes) then rinsed before cell addition.

For live cell imaging, HEK293 cells stably expressing β1Int-HaloTag, or wildtype HEK293 as a control, were labeled with the HaloTag PEG-Biotin ligand, then rinsed, and collected with 3 mM EDTA. Cells were placed into SA-coated 96 well plates (Pierce) for 30 minutes at 37°C, then wells were rinsed to remove non-captured cells. Captured cells were removed using trypsin-EDTA (Sigma), replated on glass slides (Nalge Nunc), and labeled after 24 hours with HaloTag TMR ligand and imaged.

### Glycosylation and timcourse analysis

To assess the glycosylation and location of separate protein pools in cells using the HaloTag technology we used HEK293 cells stably expressing the β1Int-HaloTag protein. Cells were labeled with the HaloTag 488 ligand alone, the TMR HaloTag ligand alone, or sequentially. Cells were then rinsed, collected in 1 × PBS with a protease inhibitor cocktail (1:100, Sigma), and lysed by fractionation. After live cell labeling and rinsing, cells were collected either immediately for the glycosylation study or at times 0–12 hours for the time course study.

Lysate was incubated with either O- or N-glycanase, using the enzymatic deglycosylation kit, per manufacturer protocol (Prozyme).

Because the HaloTag ligand is held by a stable covalent bond, the fluorescently labeled β1Int-HaloTag protein can be boiled with sample buffer and resolved by SDS-PAGE without loss of the fluorescence signal. Lysate in sample buffer (1% SDS, 10% glycerol, and 1.0 mM β-mercaptoethanol, pH 6.8) was boiled for 5 minutes and then resolved on SDS-PAGE (4–20% gradient gels, Invitrogen), with the DyLight fluorescent protein molecular weight marker (Pierce). Gels were analyzed on a fluorescence imager Typhoon 9400 (Hitachi, Japan) at an E_ex_/E_em _appropriate for Alexa Fluor^® ^488 and TMR.

## Competing interests

Authors receive salary from an organization that may be affected by the publication of this manuscript. There is one issued and two pending patents on the HaloTag^® ^technology.

## Authors' contributions

SS designed, coordinated and performed experiments, analyzed data and prepared the manuscript. CZ cloned the β1Int-HaloTag and HaloTag-(NLS)_3 _constructs and generated cells stably expressing HaloTag-(NLS)_3_. MM and DK designed and generated the HaloTag ligands. GL conceived of the study, participated in its design and critically reviewed the manuscript. All authors read and approved the final manuscript.

## Supplementary Material

Additional file 1 Distinguishing cell surface and internalized HaloTag protein. Pre trypsin exposure, live HEK293 cells sequentially labeled with HaloTag 488 and TMR ligands show spatial separation of the green surface protein around the red internal protein, and some of the original green surface pool has internalized. After trypsin, the external HaloTag 488 ligand is stripped over 200 and 400 seconds and the internal HaloTag TMR ligand and internalized HaloTag 488 ligand are preserved. Cell images were generated on an Olympus FV500 confocal microscope in sequential mode using appropriate filter sets. Click here for file

Additional file 2 Real-time translocation of proteins using HaloTag Technology. Timelapse imaging of live U2OS cells sequentially labeled with HaloTag 488 and TMR ligands shows real-time translocation of both protein pools. After labeling, time 0 shows spatial separation of the green surface protein around the red internal protein. Imaging every 20 minutes over 17 hours first shows the green surface pool internalizing and the red internal pool trafficking to the surface, then the original green surface pool inside and the red pool on the membrane, and finally the red surface pool internalizing. Click here for file
