# Supplementary figures and images for: Spatial separation and bidirectional trafficking of proteins using a multi-functional reporter
Source: BMC Cell Biol. 2008 Apr 2;9:17. doi: 10.1186/1471-2121-9-17 (PMC2359743; doi:10.1186/1471-2121-9-17)

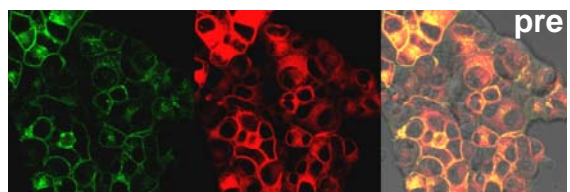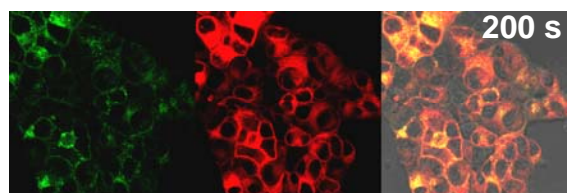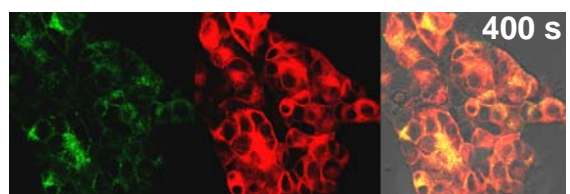

Supplement: Additional file 1 — Distinguishing cell surface and internalized HaloTag protein. Pre trypsin exposure, live HEK293 cells sequentially labeled with HaloTag 488 and TMR ligands show spatial separation of the green surface protein around the red internal protein, and some of the original green surface pool has internalized. After trypsin, the external HaloTag 488 ligand is stripped over 200 and 400 seconds and the internal HaloTag TMR ligand and internalized HaloTag 488 ligand are preserved. Cell images were generated on an Olympus FV500 confocal microscope in sequential mode using appropriate filter sets. [file 1471-2121-9-17-S1.pdf]
